# Supplementary material for: The aryl hydrocarbon receptor and interferon gamma generate antiviral states via transcriptional repression
Source: eLife. 2018 Aug 22;7:e38867. doi: 10.7554/eLife.38867 (PMC6120754; doi:10.7554/eLife.38867)
Supplement: Supplementary file 3. [file elife-38867-supp3.docx]

**(i) Pathway analysis of top 100 downregulated genes in AhR activated macrophages**

| **Pathway name** | **#Entities found** | **Submitted entities found** |
| --- | --- | --- |
| Immune System | 59 | IFITM3;IFITM1;IL1RN;IFITM2;TNFAIP6;CD80;UBE2L6;KIF11;IFI35;IFIT1;CXCL2;IFIT3;IFIT2;OASL;TNFSF13B;SOX2;IFIH1;HERC5;CDC20;MT2A;TRIM5;MYC;  DHX58;FBXO7;TRIM22;HERC6;GBP5;RSAD2;DDX58;SP110;AIM2;OAS1;IFI27;OAS2;OAS3;IRF7;LAP3;CCL3L1;CCL3L3;IFI6;USP18;CCL3;GBP1;GBP4;VCAM1;  SIGLEC14;JUP;STAT1;UBE2C;STAT2;MX2;MX1;EIF2AK2;ISG15;SOD2;ISG20;P2RX7;CXCL10;XAF1 |
| Cytokine Signaling in Immune system | 47 | IFITM3;IFITM1;IL1RN;IFITM2;CCL3L1;CCL3L3;CD80;IFI6;UBE2L6;IFI35;IFIT1;CXCL2;USP18;IFIT3;IFIT2;OASL;TNFSF13B;SOX2;HERC5;MT2A;TRIM5;MYC;  CCL3;GBP1;GBP4;TRIM22;HERC6;GBP5;VCAM1;RSAD2;DDX58;STAT1;SP110;STAT2;MX2;MX1;EIF2AK2;ISG15;SOD2;ISG20;CXCL10;OAS1;IFI27;OAS2;  OAS3;IRF7;XAF1 |
| Cell Cycle | 43 | TOP2A;FEN1;PCNA;MCM7;CDCA5;PRIM1;GMNN;NCAPG;MCM10;HMMR;TYMS;AURKB;CDC20;CCNB2;EXO1;MYC;CHEK1;E2F2;OIP5;TK1;CDC45L;FBXO5;  C16ORF75;BUB1;PLK4;CDT1;GINS2;RFC4;UBE2C;NDC80;CDC25A;CDC2;CCNA2;CCNE2;POLE2;CENPM;MCM4;MCM5;MCM6;HIST1H4C;SPC25;MAD2L1;  MCM2 |
| Cell Cycle, Mitotic | 39 | TOP2A;FEN1;PCNA;MCM7;CDCA5;PRIM1;GMNN;NCAPG;MCM10;HMMR;TYMS;AURKB;CDC20;CCNB2;MYC;E2F2;TK1;CDC45L;FBXO5;BUB1;PLK4;CDT1;  GINS2;RFC4;UBE2C;NDC80;CDC25A;CDC2;CCNA2;CCNE2;POLE2;CENPM;MCM4;MCM5;MCM6;HIST1H4C;SPC25;MAD2L1;MCM2 |
| Interferon Signaling | 36 | IFITM3;IFITM1;IFITM2;IFI6;UBE2L6;IFI35;IFIT1;USP18;IFIT3;IFIT2;OASL;HERC5;MT2A;TRIM5;GBP1;GBP4;TRIM22;HERC6;GBP5;VCAM1;RSAD2;SP110;  DDX58;STAT1;MX2;STAT2;MX1;EIF2AK2;ISG15;ISG20;OAS1;IFI27;OAS2;OAS3;IRF7;XAF1 |
| Signal Transduction | 27 | CCL13;CXCL9;CCL4L1;CD80;CCL4L2;CXCL2;AURKB;SOX2;CDC20;MYC;CCRL2;CHEK1;TNFSF10;BUB1;DLGAP5;JUP;ARHGEF15;STAT1;NDC80;CDC2;  CXCL10;CXCL11;PRC1;CENPM;HIST1H4C;SPC25;MAD2L1 |
| Cell Cycle Checkpoints | 25 | MCM7;MCM10;AURKB;CDC20;CCNB2;EXO1;CHEK1;CDC45L;C16ORF75;BUB1;RFC4;UBE2C;NDC80;CDC25A;CDC2;CCNA2;CCNE2;CENPM;MCM4;MCM5;  MCM6;HIST1H4C;SPC25;MAD2L1;MCM2 |
| Interferon alpha/beta signaling | 23 | IFITM3;IFITM1;IFITM2;RSAD2;STAT1;MX2;STAT2;MX1;IFI6;ISG15;IFI35;IFIT1;USP18;IFIT3;IFIT2;OASL;ISG20;OAS1;IFI27;OAS2;OAS3;IRF7;XAF1 |
| Mitotic G1-G1/S phases | 21 | TOP2A;CDT1;PCNA;MCM7;PRIM1;MCM10;TYMS;CDC25A;CDC2;CCNA2;CCNE2;MYC;POLE2;E2F2;MCM4;MCM5;TK1;CDC45L;MCM6;FBXO5;MCM2 |
| Metabolism of proteins | 20 | TOP2A;PCNA;UBE2C;SP110;DDX58;UBE2L6;USP18;AURKB;CDC25A;CDC2;IFIH1;CDC20;CCNA2;CCNE2;MYC;UBE2T;HIST1H4C;FBXO7;SPC25;SNCA |
| G1/S Transition | 19 | CDT1;PCNA;MCM7;PRIM1;MCM10;TYMS;CDC25A;CDC2;CCNA2;CCNE2;MYC;POLE2;MCM4;MCM5;TK1;CDC45L;MCM6;FBXO5;MCM2 |
| S Phase | 19 | CDT1;GINS2;FEN1;PCNA;RFC4;MCM7;PRIM1;CDCA5;CDC25A;CDC2;CCNA2;CCNE2;MYC;POLE2;MCM4;MCM5;CDC45L;MCM6;MCM2 |
| DNA Repair | 19 | POLQ;FEN1;PCNA;RFC4;UBE2L6;ISG15;CDC2;UNG;MSH6;RAD51AP1;CCNA2;KIAA0101;EXO1;POLE2;UBE2T;CHEK1;TIMELESS;HIST1H4C;C16ORF75 |
| DNA Replication | 18 | CDT1;GINS2;FEN1;PCNA;RFC4;MCM7;PRIM1;GMNN;MCM10;CDC2;CCNA2;POLE2;E2F2;MCM4;MCM5;CDC45L;MCM6;MCM2 |
| Post-translational protein modification | 16 | TOP2A;PCNA;UBE2C;SP110;DDX58;USP18;AURKB;CDC25A;CDC2;IFIH1;CDC20;CCNA2;MYC;UBE2T;HIST1H4C;FBXO7 |
| Metabolism | 16 | FEN1;HMMR;TYMS;PARP9;PARP14;HSPE1;NT5C3;CDC2;PRIC285;IL4I1;MARCKS;AIM2;PPAP2B;HRASLS3;TK1;PFKM |
| Gene expression (Transcription) | 15 | PCNA;RFC4;UHRF1;STAT1;ATAD2;AURKB;CDC2;PRIC285;CCNA2;CCNE2;EXO1;MYC;CHEK1;HIST1H4C;C16ORF75 |
| Synthesis of DNA | 15 | CDT1;GINS2;FEN1;PCNA;RFC4;MCM7;PRIM1;CDC2;CCNA2;POLE2;MCM4;MCM5;CDC45L;MCM6;MCM2 |
| G2/M Checkpoints | 15 | RFC4;MCM7;MCM10;CDC25A;CDC2;CCNB2;EXO1;CHEK1;MCM4;MCM5;CDC45L;MCM6;HIST1H4C;C16ORF75;MCM2 |
| M Phase | 15 | PLK4;UBE2C;CDCA5;NCAPG;AURKB;NDC80;CDC2;CDC20;CCNB2;CENPM;FBXO5;HIST1H4C;BUB1;SPC25;MAD2L1 |

**(ii) Pathway analysis of top 100 downregulated genes in IFN-γ stimulated macrophages**

| **Pathway name** | **#Entities found** | **Submitted entities found** |
| --- | --- | --- |
| Cell Cycle | 30 | TOP2A;MCM7;CDCA5;NCAPG;MCM10;HMMR;TYMS;CDC20;CCNB2;CCNB1;CCND2;EXO1;CEP70;E2F2;OIP5;NEK2;TK1;CDC45L;BUB1;GINS2;CDKN2B;UBE2C;CDC2;CCNA2;CCNE2;CENPM;MCM4;HIST1H4C;MAD2L1;MCM2 |
| Signal Transduction | 30 | RGS18;NOTCH3;EBI2;PDE3B;FAM13A;CXCR5;CXCR4;LPL;LFNG;CDC20;RGS2;NCK2;PDK4;MYH10;BUB1;DLGAP5;CDKN2B;OPN3;VWF;ITGA3;USP2;AKR1C3;CDC2;PRC1;GPER;CENPM;SDC1;UTS2;HIST1H4C;MAD2L1 |
| Cell Cycle, Mitotic | 28 | TOP2A;MCM7;CDCA5;NCAPG;MCM10;HMMR;TYMS;CDC20;CCNB2;CCNB1;CCND2;CEP70;E2F2;NEK2;TK1;CDC45L;BUB1;GINS2;CDKN2B;UBE2C;CDC2;CCNA2;CCNE2;CENPM;MCM4;HIST1H4C;MAD2L1;MCM2 |
| Metabolism | 22 | PNPLA7;CERK;ABCC5;AKR1C3;LPL;XYLT1;C7ORF68;HMMR;TYMS;BRI3BP;CDC2;NUDT7;AMDHD1;ALOX5AP;GPD1;PDK4;ACOT2;SMS;ITGB1BP3;SDC1;TK1;TNFRSF21 |
| Immune System | 20 | COLEC12;ATP8B4;TNFSF14;CLEC12A;UBE2C;KIF11;RAP1GAP;CDC20;SYNGR1;FSCN1;CD300LB;SDC1;OLR1;METTL7A;CD14;TLR5;AMICA1;CAMP;C19ORF59;HSPA1A |
| Cell Cycle Checkpoints | 17 | MCM7;UBE2C;MCM10;CDC2;CDC20;CCNA2;CCNB2;CCNB1;CCNE2;EXO1;CENPM;MCM4;CDC45L;HIST1H4C;BUB1;MAD2L1;MCM2 |
| Mitotic G1-G1/S phases | 15 | TOP2A;CDKN2B;MCM7;MCM10;TYMS;CDC2;CCNA2;CCNB1;CCND2;CCNE2;E2F2;MCM4;CDC45L;TK1;MCM2 |
| Gene expression (Transcription) | 14 | BNIP3L;NOTCH3;CDKN2B;GADD45A;USP2;TDRD9;CDC2;CCNA2;CCNB1;CCND2;CCNE2;EXO1;HIST1H4C;PHF19 |
| M Phase | 13 | UBE2C;CDCA5;NCAPG;CDC2;CDC20;CCNB2;CCNB1;CEP70;CENPM;NEK2;HIST1H4C;BUB1;MAD2L1 |
| Generic Transcription Pathway | 12 | CCNA2;BNIP3L;NOTCH3;CDKN2B;CCNB1;CCND2;CCNE2;EXO1;GADD45A;USP2;HIST1H4C;CDC2 |
| RNA Polymerase II Transcription | 12 | CCNA2;BNIP3L;NOTCH3;CDKN2B;CCNB1;CCND2;CCNE2;EXO1;GADD45A;USP2;HIST1H4C;CDC2 |
| GPCR downstream signaling | 12 | RGS18;RGS2;OPN3;EBI2;PDE3B;GPER;CXCR5;AKR1C3;SDC1;CXCR4;UTS2;LPL |
| Signaling by GPCR | 12 | RGS18;RGS2;OPN3;EBI2;PDE3B;GPER;CXCR5;AKR1C3;SDC1;CXCR4;UTS2;LPL |
| G1/S Transition | 11 | CCNA2;CCNB1;MCM7;CCNE2;MCM4;MCM10;CDC45L;TK1;TYMS;MCM2;CDC2 |
| Mitotic Prometaphase | 11 | CDC20;CCNB2;CCNB1;CDCA5;CEP70;NCAPG;CENPM;NEK2;BUB1;CDC2;MAD2L1 |
| Innate Immune System | 11 | SYNGR1;ATP8B4;CLEC12A;CD300LB;OLR1;METTL7A;CD14;TLR5;CAMP;C19ORF59;HSPA1A |
| Metabolism of lipids | 10 | NUDT7;CERK;PNPLA7;GPD1;ALOX5AP;ACOT2;AKR1C3;C7ORF68;BRI3BP;TNFRSF21 |
| G2/M Checkpoints | 10 | CCNB2;CCNB1;MCM7;EXO1;MCM4;MCM10;CDC45L;HIST1H4C;CDC2;MCM2 |
| Hemostasis | 10 | SPARC;VWF;ITGA3;PDE3B;SDC1;OLR1;TIMP3;KIF11;AMICA1;TFPI |
| Metabolism of proteins | 10 | CDC20;TOP2A;CCNA2;CCNE2;UBE2C;UBE2T;USP2;HIST1H4C;CDC2;DCAF6 |
